# Is Helping Really Helping? Health-Related Quality of Life after TBI Predicting Caregiver Depression Symptom Trajectories in Latin America

**DOI:** 10.3390/ijerph18031247

**Published:** 2021-01-30

**Authors:** Chimdindu Ohayagha, Paul B. Perrin, Annahir N. Cariello, Juan Carlos Arango-Lasprilla

**Affiliations:** 1 Department of Psychology, Virginia Commonwealth University, Richmond, VA 23284, USA; ohayaghac@mymail.vcu.edu (C.O.); pperrin@vcu.edu (P.B.P.); carielloa@mymail.vcu.edu (A.N.C.); 2 BioCruces Bizkaia Health Research Institute, 40903 Barakaldo, Spain; 3 IKERBASQUE, Basque Foundation for Science, 48009 Bilbao, Spain; 4 Department of Cell Biology and Histology, University of the Basque Country (UPV/EHU), 48940 Leioa, Spain

**Keywords:** traumatic brain injury, caregivers, Latin America, depression, health-related quality of life

## Abstract

Previous research connecting health-related quality of life (HRQoL) in people with traumatic brain injury (TBI) and caregiver mental health has primarily been conducted cross-sectionally in the U.S. and Western Europe. This study, therefore, examined how HRQoL in individuals immediately after their TBI predicts longitudinal caregiver depression symptom trajectories in Latin America. A sample of 109 patients with an acute TBI and 109 caregivers (total *n* = 218) was recruited from three hospitals in Mexico City, Mexico, and in Cali and Neiva, Colombia. TBI patients reported their HRQoL while they were still in hospital, and caregivers reported their depression symptoms at the same time and at 2 and 4 months later. Hierarchal linear models (HLM) found that caregiver depression symptom scores decreased over time, and lower patient mental health and pain-related quality of life at baseline (higher pain) predicted higher overall caregiver depression symptom trajectories across the three time points. These findings suggest that in Latin America, there is an identifiable relationship between psychological and pain-related symptoms after TBI and caregiver depression symptom outcomes. The results highlight the importance of early detection of caregiver mental health needs based in part upon patient HRQoL and a culturally informed approach to rehabilitation services for Latin American TBI caregivers.

## 1. Introduction

### 1.1. Traumatic Brain Injury in the U.S. and Latin American Countries

Traumatic brain injury (TBI) is one of the major contributors to death and disability around the world [1]. TBI is defined as an alteration to brain functioning as a result of externally applied forces [2]. The extent to which the pattern of damage is assessed is informed by the external mechanical force, its nature, direction, intensity, and duration [3]. Secondary damage, or delayed non-mechanical damage, typically involves pathological processes initiated after the impact that can result in delayed clinical presentations [4]. Secondary damage to the brain often includes cerebral ischemia, intracranial hypertension, increased intracranial pressure, hypoxia, oxidative stress, ecotoxicity, and apoptosis [5]. Common complications include neurological atrophy, neuroendocrine abnormalities, sensory disorders, fatigue, insomnia, and posttraumatic seizure disorders [6,7]. TBI can be a life-altering experience, with an estimated annual occurrence in the U.S. of 1.4 million people [8], or 500–800 new cases per 100,000 people each year [9]. Falls, motor vehicle accidents, assaults, and sports-related accidents are common causes of TBI [8], and many TBI survivors often face long-term disability [10]. Lower- and middle-income countries (LMICs) such as many Latin American countries have the highest incidence of intracranial injury worldwide [11]. Contrary to the U.S., the primary mechanisms of injury in LMICs are road traffic injuries and violence [11,12]. Recent estimates suggest that there are approximately 909 new cases of TBI per 100,000 people in LMICs each year [9]. In Mexico, TBI is ranked as the third leading cause of death, often resulting from motor vehicle accidents [13]. In Colombia, hostile guerillas and landmine explosions are also major contributors to TBI [14] with a TBI prevalence rate of approximately 6.4 per 1000 people [15].

Although TBI is a major contributor to death and disability in the U.S., according to the World Health Organization, more than 90% of deaths caused by a TBI occur in LMICs [16], where 85% of the world population lives [17]. Elevated rates of TBI are often due to living below the poverty line, residing in a conflict zone [16], a lack of preventative measures, and having less developed health systems to address physical and mental health outcomes [12,18]. Cultural and economic inequities are also significant contributors to TBI [19]. Despite the high rates of death after TBI in Latin America, there is a dearth of research in the literature or data addressing the burden of TBI in Latin America, which could limit the implementation of comprehensive TBI prevention and rehabilitation programs in the region [12].

### 1.2. Functional Outcomes Following TBI

People with TBI experience physical, behavioral, emotional, and cognitive difficulties that can persist many years after injury [20,21] and affect many aspects of everyday living, including independence, mobility, employment, and community integration [22]. Social and interpersonal skills are among the most notable impairments and have been shown to interfere with community living, occupational status, and sustainment of interpersonal relationships [23,24]. 

### 1.3. Health-Related Quality of Life Following TBI

Return to productive activity is a primary rehabilitation goal for many individuals with TBI, although complete restoration to pre-injury functioning seldomly occurs, often leaving individuals with TBI with reductions in perceived quality of life [25,26]. Health-related quality-of-life (HRQoL) is a global index of overall quality of life and is defined as an individual’s satisfaction with the physical, psychological, and social provinces of life grounded in one’s self-concept and self-efficacy, in addition to other aspects traditionally not categorized as health, such as quality of the environment, independence, and social support [27,28,29]. The evaluation of HRQoL is primarily captured through the self-report of an individual with TBI.

Several studies have shown that individuals with TBI report lower HRQoL than the general population [29,30,31]. Arango-Lasprilla and colleagues [32] investigated HRQoL in individuals with TBI living in Colombia and found that after adjusting for depression, socioeconomic status, social support, and cognitive performance, those with TBI scored poorer than healthy controls on a variety of HRQoL domains such as emotional functioning, physical functioning, and pain. These reductions often impact relationships with family members and caregivers, resulting in increased burden in caregivers [33,34].

### 1.4. Caregiver Mental Health Following Traumatic Brain Injury

People with TBI often require supervision and support from caregivers [34]. Caregiving can have positive effects on a caregiver’s well-being, such as developing a sense of strength when confronting adverse circumstances, providing a sense of accomplishment, and increasing emotional intimacy with the care recipient [35]. However, the physical, emotional, and cognitive demands of caregiving can surpass caregivers’ capacity to adequately adjust to the novel role of caregiving [34,36]. Caregivers have been shown to experience increased depression [37,38,39,40]. Arango-Lasprilla and colleagues [41] found that TBI caregivers in Colombia reported emotional support as one of the most salient unmet needs, and unmet needs have been shown to be closely related to TBI caregiver mental health problems in Mexico [42]. Similarly, Stevens and colleagues [43] found that Colombian caregivers reported being overwhelmed by caretaking responsibilities. Compared to non-caregivers, caregivers have an increased risk for depression and overall poor health [37,38], and within LMICs like those in Latin America, caregiver mental health issues could be more widespread. 

### 1.5. Current Study

Some studies have shown that patient HRQoL after TBI impacts caregiver depression [44,45], while others have identified cognitive, behavioral, and emotional changes as strong predictors of caregiver depression [46]. TBI is a family experience, impacting all members of the family. Within Latin American cultures, the familial unit is central to many aspects of life, and with the responsibility of caregiving placed upon family members, caregivers are often unprepared and lack the experience and knowledge necessary to provide comprehensive, ongoing care [47]. The mental health of TBI caregivers is paramount in terms of potentially affecting the quality of informal care provided to people with TBI. The TBI literature has shown that caregiver depression can be influenced by a myriad of factors. Harris and colleagues [34] reported that caregivers’ appraisal of adverse family effects mediated the relationship between stressors and depression, while social support moderated the relationship between adverse family effects and depression; 46% of the variance in caregiver depression was accounted for by caregivers’ appraisal of adverse family effects and social support. Linn and Willer [48] examined brain-injured patients and their spousal caregivers and found that depression was the predominant outcome, with 73% of spousal caregivers and 70% of patients exceeding the scale cutoff for depressive symptoms; the study also indicated that female spouses reported higher levels of depression compared to male spouses. Despite these findings, few studies have examined the connections between TBI patient HRQoL and caregiver depression symptoms, especially within LMICs like many in Latin America. As a result, this study aimed to examine which patient HRQoL domains predict caregiver depression symptoms over the first 4 months after injury in two countries and three hospitals in Latin America. 

## 2. Method

### 2.1. Participants

A sample of 109 patients with a new TBI and their caregivers (total *n* = 218) was recruited from three hospitals in Mexico City, Mexico, and in Cali and Neiva, Colombia. Individuals with TBI met the following inclusion criteria: (a) have a physician-confirmed diagnosis of moderate or severe TBI (Glasgow Coma Scale <13) in their medical record, (b) be at least age 18, (c) be able to communicate in Spanish, (d) be a previous or current patient at the referring center, and (e) be willing to participate with their caregiver. Caregivers met the following inclusion criteria: (a) must be the primary caregiver providing care for the person with TBI, (b) be related to the person with TBI directly via blood or marriage and/or be a close friend, (c) live in the household with the individual with TBI, and (d) be able to communicate in Spanish. Caregivers must also have had no previously documented history of severe psychological or neurological problems. Participant demographics appear in Table 1. 

### 2.2. Measures

#### 2.2.1. Short Form Health Survey

The Spanish version of the Medical Outcomes Study Short-Form Health Survey (SF-36) was utilized to measure HRQoL. The SF-36 is a well-validated instrument for measuring health status and outcomes from the patient’s perspective and has been widely used among people with TBI [49,50]. The SF-36 consists of 36 items that yield a profile of eight multi-item subscales that assess the following dimensions of health: (1) physical function, (2) physical role, (3) bodily pain, (4) general health, (5) energy/vitality, (6) social function, (7) emotional role, and (8) mental health. The eight subscales fall under two overarching categories: physical health and mental health. Scores range from 0 to 100, with higher scores reflecting greater HRQoL. The Spanish version of the SF-36 has well-established reliability and validity [32,51].

#### 2.2.2. Patient Health Questionnaire-9 

The Patient Health Questionnaire-9 (PHQ-9) depression scale is a well-validated, Diagnostic and Statistical Manual of Mental Disorders—Fourth Edition (DSM-IV) criterion-based measure for assessing depression symptoms, severity of symptoms, and monitoring treatment response [52]. The PHQ-9 maintains comparable sensitivity and specificity without the need for a two-step questionnaire to assess diagnostic criteria for depressive symptomatology, yet contains significantly fewer items among other depression symptom scales [53]. The PHQ-9 consists of 9 items and can be entirely completed by the patient, as it directs the respondent to indicate how often he/she has been bothered by each item using a response from 0 (not at all) to 3 (nearly every day). Total score ranges from 0 to 27, with higher scores indicating higher depression symptom severity. The Spanish version of the PHQ-9 has well-established reliability and validity in assessing depression symptoms within Spanish-speaking populations [54,55].

### 2.3. Procedure

Informed consent, questionnaires, and all other study materials were approved by the Virginia Commonwealth University Institutional Review Board, in addition to approval from the ethics committees at each site in Latin America. A detailed review of the patient’s records was conducted to assess if the patient (and later the caregiver) met preliminary eligibility and full inclusion/exclusion criteria. All data collections were conducted by psychologists or highly trained research assistants, either at the primary sites or in-home, depending upon the needs and preferences of the participants.

## 3. Results

### 3.1. Preliminary Analyses

A correlation matrix among the primary study variables is presented for reference in Table 2. In terms of the longitudinal analyses, one participant dropped out of the study at 2 months and four participants at 4 months. Full information maximum likelihood (FIML) estimation was used to account for missing data. Two separate main effects hierarchical linear models (HLMs) were used to examine baseline patient physical and mental HRQoL predictors of caregiver depression symptom trajectories across baseline, 2 months, and 4 months. Unconditional growth linear (straight line) and quadratic (*U*-shaped) models were run first with no predictors to determine the most accurate model for linear or polynomial curvature of caregiver depression symptom scores over time. The -2 log likelihood (-2LL) of the unconditional growth model with linear time was 1778.96, whereas the -2LL of the unconditional growth model with the addition of quadratic time was 1775.96, which represented a drop of 3.01 that did not surpass the critical 𝜒^2^ value of 3.84 for statistical significance (*p* < 0.05). As a result, the movement of caregiver depression symptom trajectories overtime was best modeled as linear.

### 3.2. Main Analyses

**Figure 1:**
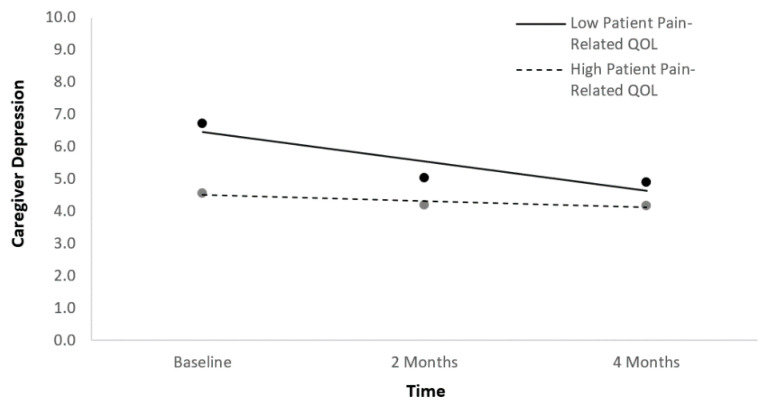
Caregiver depression symptom trajectories as a function of patient pain-related quality of life.

In the first main effects HLM predicting caregiver depression symptom trajectories, time and the four indices of patient physical HRQoL at baseline were entered simultaneously as fixed effects after being centered. All statistically significant and non-significant fixed effects from the HLM and their b-weights, *p*-values, standard errors (*SE*s), and 95% confidence intervals appear in Table 3. Time and pain yielded statistically significant effects. Caregiver depression symptom scores decreased over time. Higher patient pain-related quality of life at baseline (lower pain) was associated with lower caregiver depression across the three time points (Figure 1). A follow-up HLM was run with time, pain-related quality of life, and the time*pain-related quality of life interaction as predictors in order to determine whether caregiver depression symptom trajectories changed differentially over time as a function of patient pain-related quality of life (Table 3). The interaction term was significant, suggesting that caregiver depression symptom scores started higher but decreased more rapidly if patients had low baseline pain-related quality of life.

**Figure 2:**
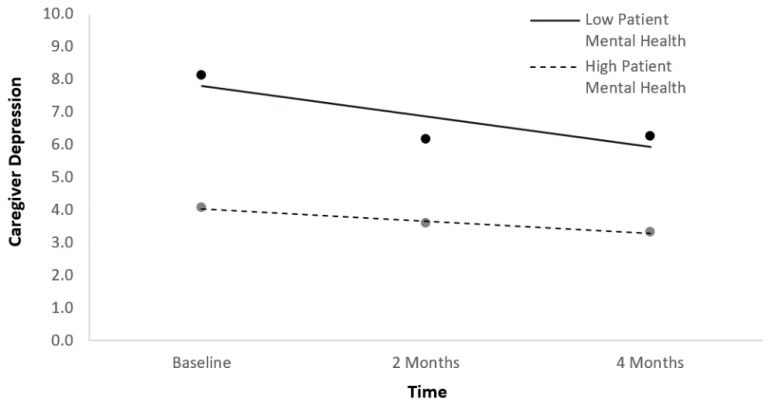
Caregiver depression symptom trajectories as a function of patient mental health.

In the second main effects HLM predicting caregiver depression symptom trajectories, time and the four indices of patient mental HRQoL at baseline were entered simultaneously as fixed effects after being centered. In the HLM, time and mental health yielded statistically significant effects such that better patient mental health at baseline was associated with lower caregiver depression symptoms across the three time points (Table 3; Figure 2). A follow-up HLM was run with time, patient mental health, and the time*mental health interaction as predictors in order to determine whether caregiver depression symptom trajectories changed differentially over time as a function of patient mental health (Table 3). The interaction term was not significant in this model, suggesting there was no differential change. 

## 4. Discussion

This study is the first to examine how HRQoL in individuals immediately after their TBI predicts longitudinal caregiver depression symptom trajectories in Latin America. Results suggested that caregiver depression symptom scores decreased over time, and lower patient mental health and pain-related quality of life at baseline (higher pain) predicted higher overall caregiver depression symptom trajectories across the three time points. There was no differential change over time in caregiver depression symptom trajectories as a function of patient mental health, though there was as a function of pain. 

Despite the improvement in caregiver depression symptoms over time, previous cross-sectional research in Mexico has found that relative to healthy age-matched controls, TBI caregivers who had been providing care for a minimum of 3 months had substantially worse satisfaction with life, self-esteem, depression, and anxiety [56], as well as lower mental HRQoL [20]. As a result, the improvement in depression symptom scores over time found in the current study is unlikely to reach levels seen in healthy controls, but nonetheless, it suggests positive psychological adjustment to their new caregiving role. 

The current study’s finding that lower pain-related quality of life (higher pain) in patients after TBI predicts higher caregiver depression symptom trajectories is unique in the literature in that the current study is the first to link pain with caregiver depression symptoms over time in Latin American countries. Given the cultural influences on TBI rehabilitation, the study also highlights TBI impairments that transcend cultures. Research suggests that 22–95% of adults with TBI experience chronic pain after injury [57,58,59]. Sullivan-Sign et al. [60] found that patient pain was the most prevalent (70%) at baseline and was significantly associated cross-sectionally with patients’ own depression. Lahz and Bryant [61] found that chronic pain occurred in 58% of mild TBI and 52% of moderate/severe TBI patients; headaches were the most commonly reported pain problem. A literature review examining the prevalence of chronic pain after TBI identified twelve studies that suggested an overall prevalence rate of 57.8% for chronic headaches in civilian and military samples [62]. A possible interpretation of these findings could be that patient pain creates a lot of psychological distress for patients which then transfers over to psychological distress for caregivers. 

Directly in line with this interpretation is the current finding that lower patient mental health at baseline predicted higher caregiver depression symptom trajectories. This finding has generally been supported by previous cross-sectional research. For example, Perrin and colleagues [63] found that brain injury patient depression was closely tied to caregiver psychosocial dysfunction in a large international sample. Stevens and colleagues [43] reported that among Spanish-speaking caregivers, caregivers’ perception of patient functioning and depression was the single best predictor of both caregiver burden and depression. Relatedly, Kreutzer and colleagues [64] found that caregivers’ perceptions of patient neurobehavioral problems were tied to caregivers’ self-report of depression; indeed, in the current study, neurobehavioral issues in patients could have been producing patient mental health problems, which then drove higher caregiver depression symptom trajectories. 

### 4.1. Clinical Implications 

Clinical assessment of patient pain and mental health while the individual with TBI is still in the hospital may inform best practices regarding treatment modalities. The larger literature has shown that pain and mental health are common co-occurring conditions [60]. It is imperative that rehabilitation clinicians evaluate potential comorbidity of pain and mental health; if patient pain is a primary variable that results in decreased mental health, then subsequent treatment for pain may lead to improvements of both mental health and pain, including caregiver mental health. Multidisciplinary interventions have been shown be most effective for pain management [65,66]. Numerous studies have confirmed that in addition to medication management, interventions such as cognitive-behavioral therapy, biofeedback, and relaxation are effective in managing pain [67]. However, the absence of psychological and mental health services may hinder patients’ ability to adhere to a treatment program effectively [68] which not only affects the patient but also the caregiver.

An integrative and culturally informed patient–caregiver approach to TBI rehabilitation would be optimal in Latin America for addressing physical consequences, such as chronic pain, and the mental health needs of both the patient and caregiver. Contrary to the fact that Latinx cultures and value systems center on collectivism and familism, TBI rehabilitation in Latin America often focuses exclusively on the individual with TBI and rarely includes interventions for caregivers [20]. Utilizing a culturally informed approach focused on the integration of caregivers during all stages of treatment is imperative given that previous studies have shown that Latin American caregivers report emotional support as a salient unmet need [41]. 

A combined patient–caregiver approach may also further strengthen cultural and familial values and lead to better rehabilitation outcomes. Familism, or familismo, refers to Latinos’ identity as part of a family [69]; it postulates that the needs of all members of a group bear greater importance than the individual’s needs, and therefore the individual sacrifices for the good of the group [70]. McCubbin and McCubbin [71] reported that families that emphasize optimism, collectivism, and their family unit, as well as shared values and goals, were more likely to positively adapt to TBI. Lehan et al. [72] found that TBI patients and caregivers who reported greater levels of family adaptability also endorsed better family communication and greater family satisfaction.

Caregiver intervention programs that are empirically supported often include stress management techniques centered on post-injury adjustment [73], psychoeducation [74], behavioral management techniques [75], and problem-solving training for family caregivers [76]. These interventions have been shown to reduce caregiver burden, anxiety, and depression [73,74,76]. Kreutzer et al. [45] investigated the benefits of a family intervention for family members of TBI patients and found that family members showed a greater number of met needs and perceived fewer obstacles to access services following intervention. When implementing these techniques to Latin America, special consideration should be given to ensure that techniques are culturally appropriate for the population, as the majority of current interventions and techniques were created for English-speaking or Western populations. 

### 4.2. Limitations and Future Directions

Although this study is among the first to examine the association between patient HRQoL and caregiver depression symptoms in Latin American TBI caregivers and patients, several notable limitations should be taken into account when interpreting the results. First, the study was conducted in two Latin American countries, and therefore, the results are not generalizable to all countries in Latin America. Latin American countries and individuals are extremely diverse, particularly with regard to socioeconomic status and rural vs. urban location. Future studies should examine whether the findings hold in other Latin American countries and regions different from those sampled in the current study. Second, although advanced trajectory modeling was utilized, study results were correlational and the analyses cannot infer causality within these relationships. Future studies should consider the use of a cross-lagged panel design to further investigate causality between patient HRQoL and caregiver depression symptoms. Third, because all measures in this study were self-report, participants likely experienced less severe TBI-related impairments compared to others with more severe impairments who, therefore, were unable to consent and participate. Caregivers of severely impaired patients may have experienced greater depression symptoms and thus may be underrepresented in the sample. Future studies should consider the combination of both self-report and objective measures of functioning. Fourth, the sample of TBI patients was overrepresented with male patients and female caregivers; caution should be taken in the generalizability of results to female patients with TBI or male caregivers. Fifth, the study consisted of a sample of relatively moderate size; future studies should recruit a larger sample from a greater number of Latin American countries. Lastly, the study only examined caregiver depression symptom trajectories at baseline, 2 months, and 4 months post-discharge. Subsequent studies should examine Latin American patient and caregiver relationships at longer time intervals, such as at 6 months, 1 year, or 5 years.

## 5. Conclusions

The current study investigated the associations among TBI patient HRQoL and caregiver depression symptoms in three sites and two countries in Latin America. The study showed that caregiver depression symptom scores decreased over time, and lower patient mental health and pain-related quality of life at baseline (higher pain) predicted higher overall caregiver depression symptom trajectories across the three time points. Because caregivers’ mental health influences the quality of informal care they can provide and is also connected to patient pain and mental health, evidence-based and culturally appropriate interventions must address the rehabilitation and mental health needs of both patients and familial caregivers.

## Figures and Tables

**Table 1:** Sample means, SDs, and percentages of demographic variables.

Caregiver Demographics		(*n* = 109)
Age, *M* (*SD*)		41.46 (13.85)
Education, years, *M* (*SD*)		10.22 (5.00)
Sex, *n* (%)	Male	20 (18.3)
	Female	89 (81.7)
Relationship to patient, *n* (%)	Parent	11 (10.1)
	Sibling	5 (4.6)
	Child	1 (0.9)
	Aunt/Uncle	1 (0.9)
	Other	41 (37.6)
Pre-injury Employment Status, *n* (%)	Employed Full-time	28 (25.7)
	Employed Part-time	22 (20.2)
	Home/Family care	34 (31.2)
	Unemployed	18 (16.5)
	Student	5 (4.6)
	Retired/Pension	2 (1.8)
Patient Demographics		(*n* = 109)
Age, *M* (*SD*)		35.87 (14.08)
Days in the hospital, *M* (*SD*)		20.78 (28.95)
Sex, *n* (%)	Male	90 (82.6)
	Female	19 (17.4)
Cause of Injury, *n* (%)	Automobile Accident	12 (11.0)
	Motor Accident	41 (37.6)
	Bicycle Accident	3 (2.8)
	Pedestrian Accident	7 (6.4)
	Firearm	2 (1.8)
	Act of Violence	17 (15.6)
	Sports Accident	1 (0.9)
	Fall	23 (21.1)
	Other	3 (2.8)
Pre-injury Employment Status, *n* (%)	Employed Full-time	34 (31.2)
	Employed Part-time	9 (8.3)
	Home/Family Care	8 (7.3)
	Unemployed	49 (45)
	Student	7 (6.4)
	Retired/Pension	2 (1.8)

**Table 2:** Correlation matrix.

Variable	1	2	3	4	5	6	7	8	9	10
1. Caregiver Depression Sx. BL										
2. Caregiver Depression Sx. 2M	0.680 **									
3. Caregiver Depression Sx. 4M	0.690 **	0.891 **								
4. Physical Functioning	−0.150	−0.170	−0.194 *							
5. Role—Physical	−0.054	0.063	0.023	0.447 **						
6. Role—Emotional	−0.232 *	−0.142	−0.121	0.250 **	0.514 **					
7. Vitality	−0.193 *	−0.168	−0.213 *	0.490 **	0.419 **	0.396 **				
8. Mental Health	−0.338 **	−0.276 **	−0.295 **	0.406 **	0.316 **	0.404 **	0.709 **			
9. Social Functioning	−0.234 *	−0.189	−0.187	0.496 **	0.381 **	0.464 **	0.560 **	0.506 **		
10. Pain	−0.266 **	−0.166	−0.143	0.506 **	0.388 **	0.310 **	0.478 **	0.375 **	0.485 **	
11. General Health	−0.128	−0.184	−0.174	0.428 **	0.286 **	0.388 **	0.529 **	0.551 **	0.444 **	0.149

Note. * = *p* < 0.05; ** = *p* < 0.01; Sx. = symptoms. BL = baseline.

**Table 3:** Predictors of caregiver depression symptom trajectories at baseline, 2 months, and 4 months based on TBI HRQoL.

Predictor	*b*-Weight	*SE*	*p*-Value	95% Confidence Interval	
				Lower	Upper
Main Effects Model 1					
Intercept	5.67	0.46	0.000	4.75	6.58
Time	−0.64	0.18	0.001	−1.00	−0.28
Physical Functioning	−0.01	0.02	0.510	−0.04	0.02
Role—Physical	0.02	0.01	0.094	−0.00	0.05
Pain	−0.03	0.02	0.041	−0.06	−0.00
General Health	−0.03	0.02	0.142	−0.07	0.01
Interaction Effects Model 1					
Intercept	5.62	0.47	0.000	40.69	6.55
Time	−0.64	0.18	0.000	−1.00	−0.29
Pain	−0.04	0.01	0.003	−0.07	−0.01
Time*Pain	0.01	0.01	0.020	0.002	0.02
Main Effects Model 2					
Intercept	5.64	0.46	0.000	4.73	6.55
Time	−0.70	0.18	0.000	−1.05	−0.34
Role—Emotional	−0.01	0.01	0.603	−0.03	0.02
Vitality	0.02	0.03	0.439	−0.03	0.07
Mental Health	−0.07	0.03	0.014	−0.12	−0.01
Social Functioning	−0.01	0.02	0.560	−0.04	0.02
Interaction Effects Model 2					
Intercept	5.62	0.46	0.000	4.71	6.52
Time	−0.64	0.18	0.000	−1.00	−0.29
Mental Health	−0.07	0.02	0.000	−0.11	−0.04
Time*Mental Health	0.01	0.01	0.168	−0.00	0.02

## Data Availability

The data presented in this study are available on request from the corresponding author. The data are not publicly available due to participant confidentiality.
